# Epidemiology of multiple sclerosis and vitamin D levels in Lanzarote, Canary Islands, Spain

**DOI:** 10.7717/peerj.8235

**Published:** 2019-12-18

**Authors:** Silvia Pérez-Pérez, Pablo Eguia del Rio, María Inmaculada Domínguez-Mozo, María Ángel García-Martínez, María Francisca Zapata-Ramos, Maria Jose Torrejon, Rafael Arroyo, Roberto Alvarez-Lafuente

**Affiliations:** 1 Grupo de Investigación de Factores Ambientales en Enfermedades Degenerativas, Instituto de Investigación Sanitaria del Hospital Clínico San Carlos (IdISSC), Hospital Clínico San Carlos, Madrid, Spain; 2 Servicio de Neurología, Hospital Doctor José Molina Orosa, Lanzarote, Spain; 3 Servicio de Análisis Clínicos, Hospital Doctor José Molina Orosa, Lanzarote, Spain; 4 Servicio de Análisis Clínicos, Instituto de Investigación Sanitaria del Hospital Clínico San Carlos (IdISSC), Hospital Clínico San Carlos, Madrid, Spain; 5 Servicio de Neurología, Hospital Universitario Quirónsalud Madrid, Madrid, Spain

**Keywords:** Multiple sclerosis, Vitamin D, Prevalence, Incidence, Lanzarote, Spain

## Abstract

**Background:**

Low levels of 25-hydroxyvitamin D (25(OH)D) have been described as one of the possible environmental factors involved in multiple sclerosis (MS) etiopathogenesis.

**Objectives:**

To study epidemiology of MS and 25(OH)D serum levels of patients in Lanzarote (29°02′06″N), a region with high ultraviolet radiation values during the whole year which is located far apart from Iberian Peninsula (36°–43°N), but without genetic/ethnic differences with it.

**Methods:**

Incidence in Lanzarote was assessed according to McDonald 2005 criteria between January 2008 and December 2015 and prevalence date was 12/31/15. For 25(OH)D serum levels analyses, samples from 60 MS patients and 60 healthy donors (HD) were collected monthly in a one-year prospective study.

**Results:**

The prevalence of MS in Lanzarote was 50.0/100,000 and the incidence per year was 2.5/100,000. Median 25(OH)D levels values were 29.1 ng/ml for MS patients (maximum = 36.1 ng/ml, minimum = 22.5 ng/ml) and 27.1 ng/ml for HD (maximum = 34.8 ng/ml, minimum = 22.8 ng/ml). There were no significant differences between 25(OH)D serum levels between MS patients and HD.

**Conclusions:**

Lanzarote possesses lower prevalence and incidence values than peninsular Spain. Moreover, 25(OH)D serum levels do not differ between MS patients and HD.

## Introduction

Multiple sclerosis (MS) is a chronic and demyelinating disease of the central nervous system (CNS). Both genetic and environmental factors seem to interplay a role in its etiopathogenesis (Handel et al., 2010). Hypovitaminosis D is one of the environmental factors related to MS (Goodin, 2014).

For many years, a latitudinal gradient has been described for MS; equatorial regions showed lower prevalence and incidence values than regions with higher latitudes (Alonso & Hernán, 2008; Simpson et al., 2011). In recent years, this gradient seems to be disappearing with a generalized increase of MS cases in all regions (longer life expectancy of the patients, better diagnosis of the disease, etc.), and regions that used to present low prevalence figures are now showing similar rates to other located at high latitudes (Koch-Henriksen & Sørensen, 2010). However, there is still an epidemiological pattern, marked by climate and lifestyle. For example, in Bulgaria, there exists a correlation between MS prevalence and climatological factors (temperature, precipitations, sunshine hours per day, etc.), instead of latitude (Kalafatova, 1987). In addition, studies with twins show that differences in the sunlight to which they are exposed are related to the discordance of the disease between them (Islam et al., 2007).

Moreover, there is increasing evidence of the involvement of hypovitaminosis D in MS pathology. The link between hypovitaminosis D and MS development, and the fact that the main source of vitamin D is its biosynthesis at epithelial cells carried out by ultraviolet B (UVB) radiation (Adams & Hewison, 2010), highlight a relationship between sunlight exposure, vitamin D and MS.

**Figure 1:**
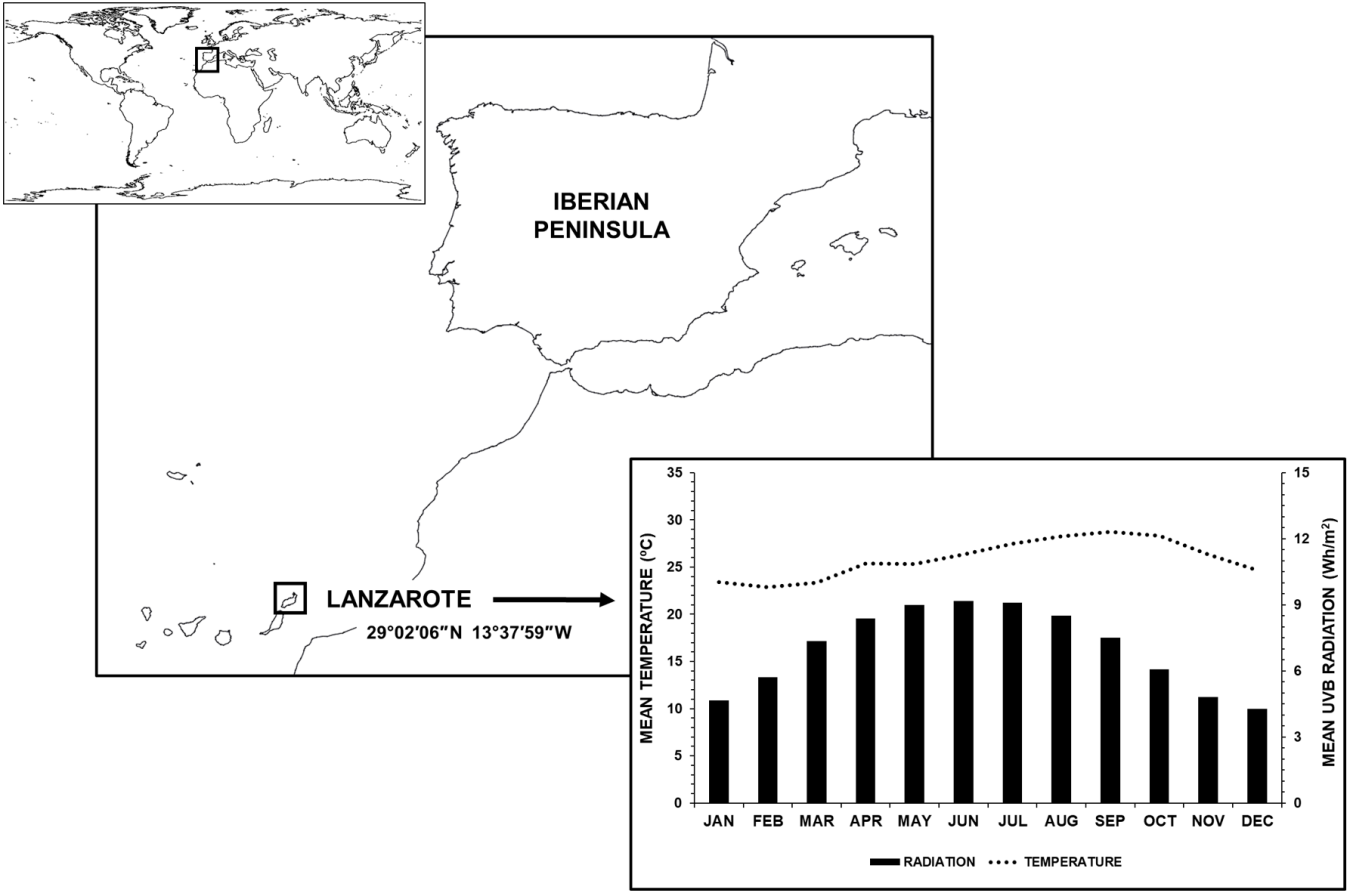
Geographic location of Lanzarote. Graphs show monthly UVB radiation rates and temperatures for both cities. Made with Natural Earth.

According to the Atlas of MS (“Atlas of MS, 2018”), in 2013 the prevalence in Spain was 100.0/100,000 and the incidence was 4.0/100,000. Iberian Peninsula is located in South-western Europe, with a latitude ranging from 36°N to 43°N. However, Canary Islands, also belonging to Spain, are located far away from this range of latitudes; in particular, Lanzarote is located at 29°N and its UVB radiation and weather are very different to those of the Iberian Peninsula (Fig. 1).

In the present article, we aimed to study the prevalence and incidence of MS in Lanzarote and to relate these data to 25(OH)D serum levels of the patients and its climatic conditions.

## Materials & Methods

### Study design and setting

We performed a prospective study of MS in Lanzarote (29°02′06″N, 13°37′59″W), the Eastern island of the Canary Islands (Fig. 1), from January 2008 to December 2015. The climate is warm (the mean temperature is 22 °C), and it has a mean annual rainfall of 140 mm^3^. This island is possesses a characteristic orography and geography, defined by flat, desert and dried fields. The total population, according to the 2015 census, was 143,209 inhabitants (70,510 women and 72,699 men), representing 6.81% of the total population of the Canary Islands (“Instituto Nacional de Estadistica, 2018. (Spanish Statistical Office)”). It has a well-established public healthcare system provided by two general hospitals and fifteen primary health care centers. The only Neurology Department is at Doctor José Molina Orosa Hospital. There are also one small private hospital and two private clinics, with two private magnetic resonance (MR) facilities.

**Figure 2:**
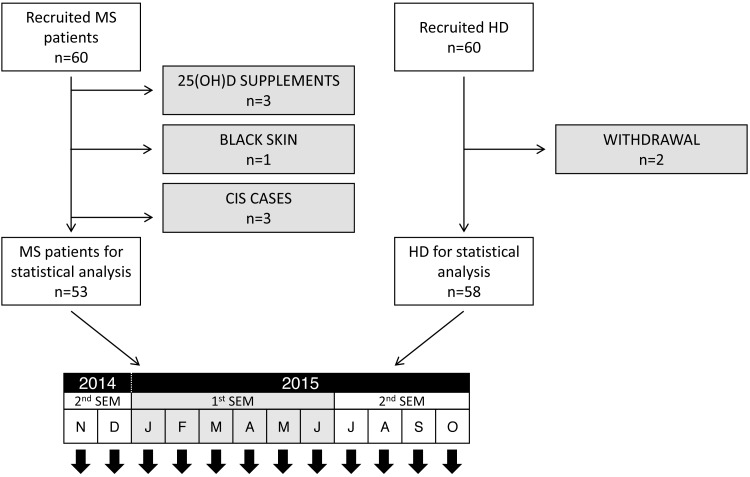
Study design for 25(OH)D analysis. A serum sample was collected monthly from each patient. Grey boxes show individuals that were excluded from the analyses (exclusion criteria: withdrawal from the study, CIS diagnoses, vitamin D supplements intake and black-coloured skin).

During this period, we carried out another parallel prospective study in which we enrolled 60 MS patients and 60 healthy donors (HD) in order to analyse vitamin D serum levels. Inclusion criteria for MS patients were their acceptance of enrolment (it was proposed to every MS patient in Lanzarote); HD were selected among the hospital staff, matched by gender and age with the enrolled MS patients. We prospectively collected serum samples monthly throughout one year, from November 2014 to October 2015. Immediately after collection, serum samples were aliquoted and stored at −80 °C until analysis. Figure 2 shows the study design; individuals who met one of the exclusion criteria (withdrawal from the study at any time, patients diagnosed with clinically isolated syndrome –CIS –, vitamin D supplements intake or being black-skin coloured) were excluded from the analyses. Table 1 shows demographical data of the included patients.

**Table 1:** Description of the basal demographical characteristics of the patients and HD included in the vitamin D substudy (at the first collected sample).

	**MS**	**HD**
*N*	53	58
Gender [*n* (%)]		
Males	19 (35.8)	19 (32.8)
Females	34 (64.2)	39 (67.2)
Age (years old, median (P25–P75))	43.0 (37.0–51.0)	43.0 (35.5–50.8)
Birhtplace [*n* (%)]		
Lanzarote	29 (54.7)	36 (62.1)
Canary Islands (not lanzarote)	5 (9.4)	7 (12.1)
Iberian Peninsula	10 (18.9)	15 (25.8)
Europe (Not Spain)	7 (13.2)	–
South America	2 (3.8)	–
MS Course [*n* (%)]		–
RR	49 (92.4)	–
SP	2 (3.8)	–
PP	2 (3.8)	–
Disease duration (months, median (P25–P75))	135.0 (77.0–200.0)	–
Current treatment [*n* (%)]		
First line therapy (interferon beta; glatiramer acetate)	17 (32.1)	–
Second line therapy (Natalizumab; Fingolimod)	12 (22.6)	–
Without treatment	24 (45.3)	–
Treatment duration (Months, median (P25–P75))	45.0 (15.0–69.0)	–
EDSS (Median (P25–P75))	1.5 (1.0–3.0)	–
Age of disease onset (years old, median (P25–P75))	30.0 (25.0–36.0)	–

### Sources of information and data collection

Cases were obtained from Doctor José Molina Orosa Hospital. We also contacted Primary Care physicians, private hospitals and clinics and regional patient associations. The study protocol was approved by the Ethics Committee for Clinical Research of the Doctor Negrín Hospital and the Medical Director of the Doctor José Molina Orosa Hospital. The study was conducted in accordance with the Declaration of Helsinki. All participants received and signed a written informed consent before enrolment.

### Variables of the study

- **Case ascertainment and definition criteria**

Incident cases were defined as confirmed new MS patients according to McDonald 2005 criteria (Polman et al., 2011) who had the onset of symptoms between January 2008 and December 2015. Prevalence of MS was calculated on December 31st 2015, including all patients living on the island but excluding deceased patients, those who had moved away from the island by that date or those who were residents on a temporary basis (defined as not registered in the census).

- **Samples and 25(OH)D determination**

Despite not being active, 25-hydroxyvitamin D (25(OH)D) is used to evaluate vitamin D serological levels (Adams & Hewison, 2010). 25(OH)D levels were analysed by chemiluminescent microparticle immunoassay (CMIA) (Abbot, Wiesbaden, Germany), following the manufacturer’s instructions. All samples were analysed in the Department of Clinical Analysis of Hospital Clínico San Carlos, at the same time, and using the same device.

- **Climatological data**

All climatological data were obtained from the SoDa Service (http://www.soda-pro.com) (“Home—http://www.soda-pro.com”). For Lanzarote (Fig. 1), we used daily UVB (280–315 nm) radiation rates and temperatures from January 1st 2008 to December 31st 2015 to calculate the mean value per month. For the rest of the countries, we calculated annual mean, maximum and minimum UVB radiation and temperature values of their capital, following the same process aforementioned.

### Statistical analysis

Prevalence and incidence rates were expressed per 100,000 inhabitants and the 95% confidence intervals (CI) were calculated assuming a Poisson’s distribution. The population was non-parametric, according to Kolmogorov–Smirnov test. Differences in 25(OH)D serum levels between MS patients and HD (median value of all the samples from each patient) were analysed using the Mann–Whitney *U* test; we also compared 25(OH)D levels in each month, taking into account that these levels vary throughout the year depending on sunlight exposure. In addition, we stratified participants by gender, age and treatment to evaluate their possible effect in 25(OH)D serum levels in our cohort. We calculated Spearman’s rank correlation coefficient for correlations between 25(OH)D serum values (median levels of each month) and UVB radiation rates corresponding to the same and the previous months (median values). Statistically significant differences were considered when *p* < 0.05. All analyses were carried out using SPSS 21.0 software (SPSS Inc., Chicago, IL, USA).

## Results

On the prevalence day, there were 71 MS patients in Lanzarote according to McDonald 2005 criteria. Therefore, on December 31st 2015, the prevalence of MS in Lanzarote was 50/100,000 (95% CI [44.4–56.2]). In the period of study (January 2008–December 2015), the incidence of MS in Lanzarote per year was 2.5/100,000 (95% CI [1.1–3.8]), that it is to say a mean of 3.5 new MS patients each year.

**Figure 3:**
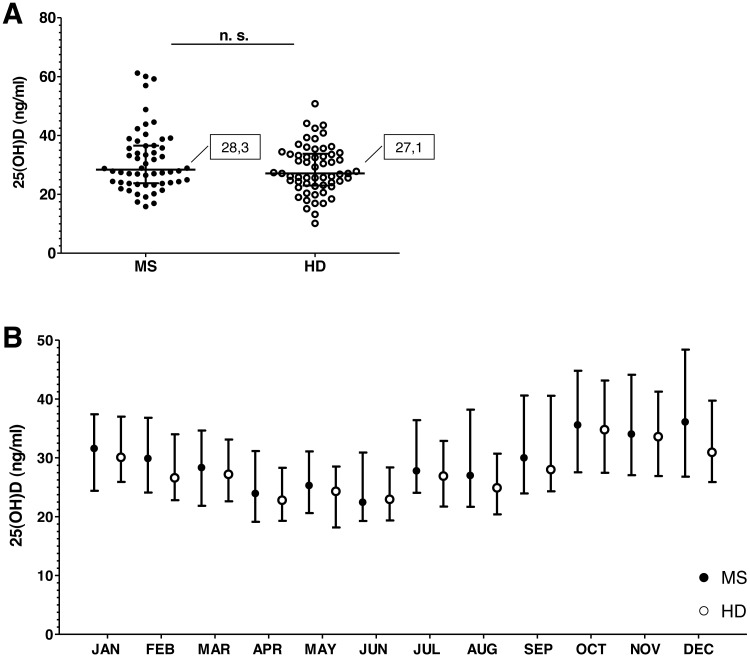
Differences in 25(OH)D serum levels between MS patients (MS) and healthy donors (HD). Graphs show differences for the whole year (A) and throughout the months (B). Dot-plot lines show the median value and the interquartile range. Statistical analysis were performed according to the Mann-Whitney *U* test.

Regarding 25(OH)D serum levels, MS patients showed higher 25(OH)D serum levels than HD (MS: median = 28.3 ng/ml, maximum monthly median (September) = 36.1 ng/ml, minimum monthly median (March) = 22.5 ng/ml; HD: median = 27.1 ng/ml, maximum monthly median (July) = 34.8 ng/ml, minimum monthly median (January) = 22.8 ng/ml), but differences between them were not statistically significant (Fig. 3A). There were neither statistically significant differences between MS patients and HD in any month. There were no significant differences in 25(OH)D serum levels in terms of gender, age or treatment (File S1).

Concerning UVB radiation rates and 25(OH)D serum levels relationship, we obtained the best correlation coefficient between serum levels of 25(OH)D and UVB radiation rates three months before, both for MS patients (*r*-value = 0.935, *p*-value = 8.32e−6) and HD (*r*-value = 0.871, *p*-value = 2.23e−4), according to the Spearman’s rank correlation coefficient.

## Discussion

In spite of belonging to Spain, Lanzarote is located far away from the Iberian Peninsula. This island presents different latitude, climatic conditions, UVB radiation rates and lifestyle than peninsular Spain. The characteristics of Lanzarote allowed us to perform an accurate calculation of prevalence and incidence of MS in this region. As aforementioned, on 31st December 2015, MS prevalence in Lanzarote was 50.0/100,000 and, from January 2008 to December 2015, the incidence was 2.5/100,000.

**Figure 4:**
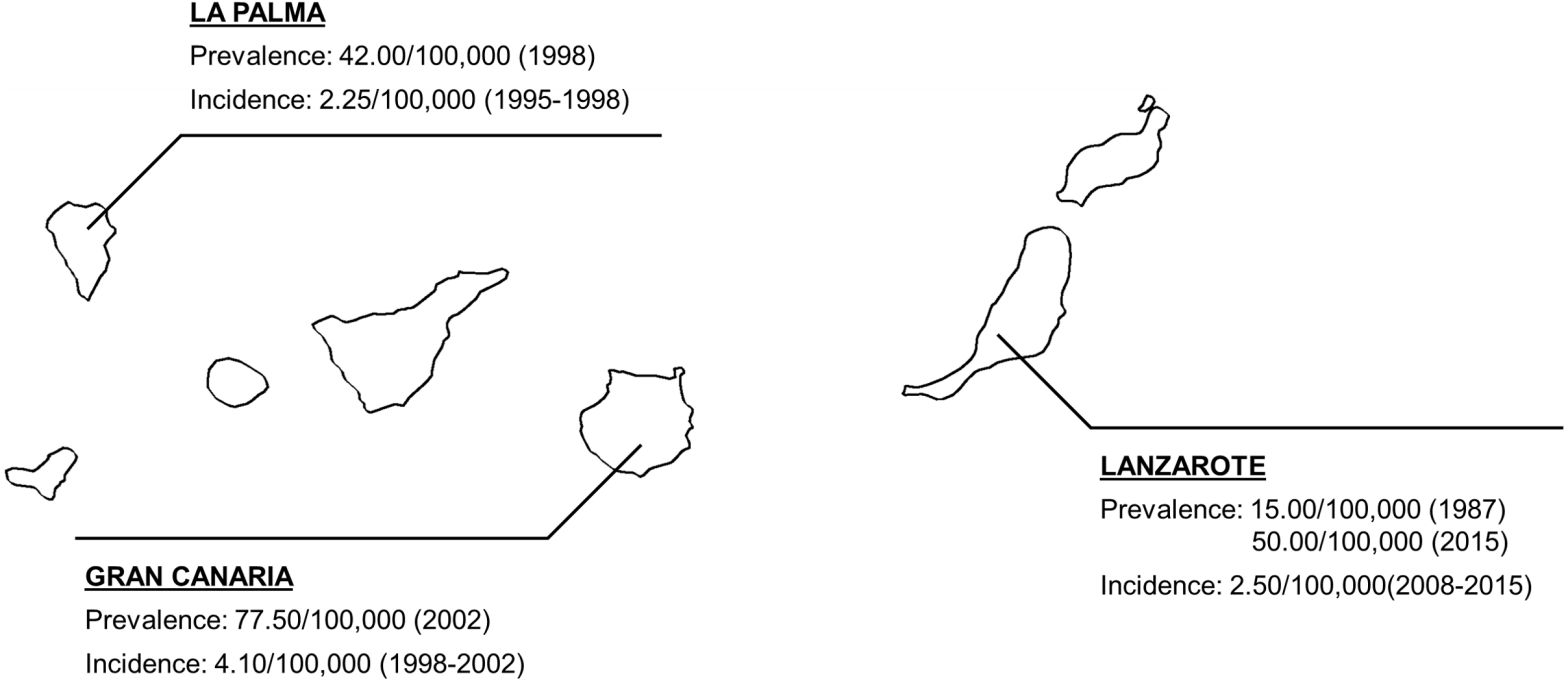
Epidemiological studies carried out in the Canary Islands. The map shows every previous epidemiological data obtained in the Canarian archipelago (Garcia et al., 1989; Hernández, 2002; Aladro et al., 2005). Made with Natural Earth.

Although Lanzarote is geographically located near to Africa, there are no genetic or ethnic differences with peninsular Spain population. As reported by the Atlas of MS (“Atlas of MS, 2018”) and the Spanish Neurological Society (“Sociedad Española de Neurología, 2018. (Spanish Neuological Society)”), the prevalence and incidence in Spain in 2013 were 100.0/100,000 and 3.8–4.0/100,000, respectively. Therefore, both prevalence and incidence were lower in Lanzarote than in peninsular Spain. Other epidemiological studies carried out in Europe reflect the variation in prevalence and/or incidence across the continent according to the latitude of the country (Kingwell et al., 2013). In Spain, several epidemiological studies have been performed throughout the last decades. Most of them took place many years ago, so we cannot compare the figures obtained by them with ours, due to the increasing prevalence of MS because of the better diagnosis of the disease or the increased life expectancy of patients. However, the latter ones have reported prevalence values of 125.0/100,000 in Málaga (Fernández et al., 2012) and 90.2/100,000 in Seville (Izquierdo et al., 2015), both located in Southern Spain. In the particular case of the Canary Islands (Fig. 4), the only study performed in Lanzarote showed a prevalence of 15.0/100,000 (Garcia et al., 1989), but it was carried out almost 30 years ago. Considering other islands of the archipelago, Hernández reported that the prevalence of MS in La Palma island was 42.0/100,000 in 1998 (Hernández, 2002), while Aladro et al. reported a prevalence value of 73.8/100,000 in Gran Canaria island in 2002 (Aladro et al., 2005).

In view of the MS epidemiology of Lanzarote, we considered a possible link to the climatological conditions and/or, consequently, to 25(OH)D levels in this region. Therefore, we evaluated 25(OH)D serum levels of MS patients and HD from Lanzarote throughout the year. We firstly included serum samples from 60 MS patients and 60 HD. However, some of these individuals were not finally included in our analyses due to one of the next reasons: (1) withdrawal from the study (two HD), (2) CIS cases, (3) vitamin D supplements intake (three MS patients) and (4) black-coloured skin (one MS patient). We excluded patients who were treated with vitamin D supplements because their 25(OH)D serum levels would not be representative and black patients since skin pigmentation correlates negatively to 25(OH)D synthesis (Clemens et al., 1982). Regarding above results, we found no differences in 25(OH)D levels between MS patients and HD, neither considering the whole year median nor monthly values. In Lanzarote, the median of 25(OH)D levels of patients was 29.1 ng/ml, reaching its maximum in October (36.1 ng/ml) and its minimum in April (22.5 ng/ml). Even the lowest median 25(OH)D levels in Lanzarote are above 20 ng/ml, the value established by the US Institute of Medicine in 2011 (Ross et al., 2011) as the threshold for acceptable 25(OH)D serum levels. In a previous study of our group performed with MS patients from Madrid (the capital of Spain, located in the centre of the Iberian Peninsula) (Pérez-Pérez et al., 2018), we described that the median of 25(OH)D levels were 20.5 ng/ml, reaching its maximum in September (26.40 ng/ml) and its minimum in March (13.30 ng/ml). Therefore, the lowest 25(OH)D value in Lanzarote is almost higher than the highest one in Madrid.

Considering climatological features of Lanzarote and Madrid, 25(OH)D levels are directly proportional not only to UVB radiation rates but also to mean temperatures. In Lanzarote, UVB radiation rates range from 4.274 to 9.183 kWh/m2; in Madrid, in spite of having a higher maximum punctual rate (9.943 kWh/m2 registered in July), there exists a huge variation among months, with rates lower than the minimum value of Lanzarote from November to February. In terms of temperature, it is practically constant throughout the year in Lanzarote (23 °C–29 °C), while Madrid shows a strong fluctuation from winter (9 °C) to summer (31 °C).

We also evaluated the relationship between UVB radiation rates and 25(OH)D serum levels in MS patients and HD. Examining 25(OH)D data of every month and UVB values of the same and the previous months, we found that the highest and more significant correlation coefficient appeared when linking 25(OH)D serum levels (MS patients or HD) and the UVB radiation values of three months before. This means that UVB radiation needs a period of action to be effective.

Among the multiple functions attributed to vitamin D, it should be pointed out its immunomodulatory characteristics in relation to MS pathology (Calton et al., 2015). The link between hypovitaminosis D and MS is broadly documented. Many studies have proposed the deficiency of vitamin D as a risk factor for MS. Munger et al. (2006) showed that high 25(OH)D levels were associated with a significantly lower risk of developing MS in a cohort of seven million US soldiers. Even during pregnancy, low 25(OH)D levels of mothers have been demonstrated to be a risk factor for MS development in their offspring (Munger et al., 2016). Low levels of vitamin D have also been related to a worse disease course (Runia et al., 2012). However, we have not found significant differences in 25(OH)D serum levels of MS patients and HD in Lanzarote. It is well known that MS is a multifactorial disease and, despite vitamin D has a role in its etiopathogenesis, it is not the only factor involved. Lanzarote could present lower prevalence and incidence values than the rest of Spain maybe due to the similar 25(OH)D serum levels between patients and healthy donors, not being hypovitaminosis D a risk factor for MS development in this place. In addition, the weather (warm temperatures, few rains, etc.), the particular arid orography and the lifestyle in Lanzarote make people to be more exposed to UV radiation. This island is not characterized by urban lifestyles and people (HD and patients) are more exposed to UV radiation. Moreover, these characteristics allow children and teenagers to spend more time outdoors (in the playground in the morning, in the street in the evening…) than the ones in peninsular Spain and, especially, in northern Europe. The sunlight exposure in early years could be crucial for avoiding MS development (Van der Mei et al., 2003; Kampman, Wilsgaard & Mellgren, 2007) and it could be interesting to reproduce this study not only in adults but also in healthy children and teenagers.

**Figure 5:**
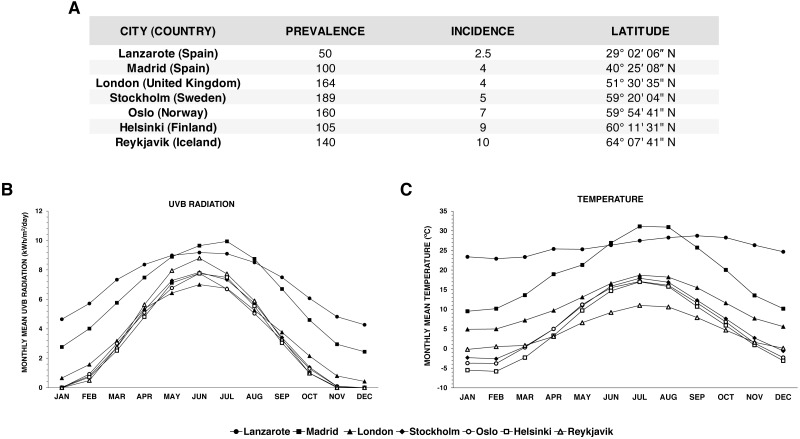
Epidemiological data for other European regions. (A) Prevalence and incidence of MS in Lanzarote and other European cities, showing their latitude. (B) Monthly fluctuation of UVB radiation rates throughout the year in those cities. (C) Monthly variation of mean temperatures in those cities. Prevalence and incidence values are expressed per 100,000 inhabitants.

It is difficult to compare epidemiological data with vitamin D levels of other European countries because there are no such studies. For this reason, and considering that the main factor that affects 25(OH)D levels is UVB radiation rate and, by extension, latitude, we have shown in Fig. 5 the latitude, the prevalence and the incidence of several European capitals (“Atlas of MS”), as well as their evolution of UVB radiation and temperature (“Home—http://www.soda-pro.com”) throughout the year. We can see that cities of Northern-Europe, with low winter UVB rates and temperatures, showed higher prevalence and incidence values than Lanzarote. It is noteworthy that, in the concrete case of the incidence, which offers a better reflection of MS epidemiology (since, on the contrary to prevalence, it is not affected by factors such as an increased life expectancy), it shows an almost perfect correlation with latitude. Previous studies have also reported a correlation between epidemiology of MS and latitude, UVB radiation and weather (Orton et al., 2011; Simpson et al., 2011; Evans et al., 2013).

It is important to highlight that we have been able to collect serum samples from almost all MS patients in Lanzarote (60 in 71), which implies that the aforementioned results are an accurate reflection of the disease on the island. As a weakness of this study, the fact that the HD cohort is made of hospital staff could have affected the results since their scientific knowledge could result in healthier habits and, consequently, in their vitamin D levels in comparison to MS patients. Everything above exposed seems to indicate two things. The first one is that a constant UVB radiation exposure, and not only high peaks followed by moments of almost no exposure, is determinant for having optimal 25(OH)D levels. The second one is that, although UVB radiation rates vary across the year, and it is not exactly the same in all months due to the different incidence angle of sunlight rays, the existence of a constant, warm climate favour being more exposed to UVB radiation during the whole year (wearing lighter clothes, spending more time outdoors, etc.) and, consequently, making the most of vitamin D synthesis. Moreover, there is evidence that supports that UVB radiation has immunomodulatory effects independent of vitamin D (Breuer et al., 2014), which points out the importance not only of having adequate vitamin D levels but also of being exposed to UVB radiation. It is important to highlight that, the weather of Lanzarote makes the inhabitants of the island to be exposed to sunlight the whole year (not only because of the sunlight rate *per se* but also for the warm climate which allows them to wear light clothes and to spend more time outdoors, being even more exposed to sunlight). Going back to the previously mentioned idea of a constant UVB radiation exposure, maybe the particular conditions of Lanzarote are the reason for having lower prevalence and incidence figures than the rest of Spain and, especially, than the rest of Europe.

## Conclusions

To sum up, latitude and UVB radiation would not be the only cause of the apparent prevalence and incidence gradient observed for MS, but to make the most of this UVB radiation, which is mainly conditioned by the lifestyle. However, further, more exhaustive studies are required to elucidate whether UV radiation, 25(OH)D serum levels or lifestyle could be responsible for the latitudinal gradient described for MS.

##  Supplemental Information

10.7717/peerj.8235/supp-1 Data S1 Demographic information and 25(OH)D levels of MS patients and HD

10.7717/peerj.8235/supp-2 File S1 Differences in 25(OH)D between groups There were no significant differences in 25 (OH)D levels in terms of gender, age, treatment or MS clinical type.

## Additional Information and Declarations

### Data Availability

The following information was supplied regarding data availability: The raw measurements are available in the Supplemental File.
