# Supplementary material for: Epidemiology of multiple sclerosis and vitamin D levels in Lanzarote, Canary Islands, Spain
Source: PeerJ. 2019 Dec 18;7:e8235. doi: 10.7717/peerj.8235 (PMC6966995; doi:10.7717/peerj.8235)
Supplement: File S1 — There were no significant differences in 25 (OH)D levels in terms of gender, age, treatment or MS clinical type. [file peerj-07-8235-s002.docx]

| **VARIABLE** | **p-value** |
| --- | --- |
| **GENDER** | 0.069 (U-Mann Whitney: MS vs HD)) |
| **AGE** | 0.068 (U-ManWhitney: >40 vs <40 ) |
| **TREATMENT** | 0.769 (Kruskal Wallis: First line vs Second line vs w/o) |
| **MS CLINICAL TYPE** | 0.125 (Kruskal Wallis: RR vs PP vs SP vs CIS*) |

*Despite there were not significant differences in 25(OH)D levels with CIS patients, we decided to exclude them because of the possibility that they do not become MS.
